# Neurocognitive Dysfunction Risk Alleviation With the Use of Dexmedetomidine in Perioperative Conditions or as ICU Sedation: A Meta-Analysis

**DOI:** 10.1097/MD.0000000000000597

**Published:** 2015-04-10

**Authors:** Bo Li, Huixia Wang, Hui Wu, Chengjie Gao

**Affiliations:** From the Department of Anesthesiology (BL, HW, CG), Jinan General Hospital, PLA Jinan Military Area Command, Jinan; and Department of Anesthesiology (HW), The People's Hospital of Zhangqiu, Zhangqiu, Shandong, China.

## Abstract

Many studies have reported the beneficial effects of dexmedetomidine on postoperative neurocognitive function but overall evidence is not as clear. We examined this conundrum by meta-analyzing studies that used dexmedetomidine in perioperative conditions or as intensive care unit (ICU) sedation and utilized reliable neurocognitive assessment tests.

The literature search was undertaken across several electronic databases including EBSCO, Embase, Google Scholar, Ovid SP, PubMed, Scopus, and Web of Science.

Literature search was carried out across several electronic databases and relevant studies were selected after following précised inclusion criteria. Meta-analysis of risk differences (RDs) was carried out and subgroup analyses were performed.

Twenty studies were selected from which data of 2612 individuals were used. Initial dexmedetomidine dose was 0.68 ± 0.27 and maintenance dose was 0.54 ± 0.32 in the trials. Dexmedetomidine treatment was associated with significantly lower risk of postoperative/postanesthesia neurocognitive dysfunction both in comparison with saline-treated controls (RD [95% confidence interval, CI]: −0.17 (−0.30, −0.04); *P* = 0.008) and comparators (−0.16 [−0.28, −0.04]; *P* = 0.009). In the subgroups analyses, however, there was no significant differences between dexmedetomidine and controls/comparators when studies with confusion assessment method for ICU only (RD: −0.10 (−0.22, 0.02); *P* = 0.1) or midazolam as comparator only (RD: −0.26 (−0.60, 0.07); *P* = 0.12) were meta-analyzed.

Dexmedetomidine use in the perioperative conditions or as ICU sedation is associated with lower risk of neurocognitive dysfunction. There can be some impact of neurocognitive assessment method, drug interactions, and clinical heterogeneity on the overall outcomes of this meta-analysis.

## Abstract 2

Supplemental Digital Content is available in the text

## INTRODUCTION

It is well-recognized that intensive care unit (ICU) survivors face a high risk for cognitive impairment that may persist much longer after recovery.^1^ Emergence delirium is an acute form of brain dysfunction that can become dangerous and result in serious consequences for the patient including injury, severity in pain, hemorrhage, and self-extubation.^2^ Such a form of neurocognitive dysfunction affects up to 80% of mechanically ventilated ICU patients and is a predictor of cognitive impairment in elderly patients without critical illness.^1^ The main risk factors for postoperative cognitive impairment and decline include increasing age, low education level, and severity as well as duration of surgery^3^; besides, preoperative benzodiazepines use and surgery type are also identified as risk factors.^2^

Dexmedetomidine is a potent, highly selective α-2 adrenoceptor agonist that mediates its effects via the G-protein in the central nervous system to inhibit sympathetic nerve firing leading to reduction in blood pressure and heart rate, sedation, and anxiolysis.^4^ In healthy young individuals, electroencephalography of sleep spindles shows that the sedative effects of dexmedetomidine resemble S2 sleep in humans.^5^ Infusion of a small dose of dexmedetomidine in healthy individuals provides sedation that is arousable with verbal commands.^6^ Dexmedetomidine manifests its effects in a dose-dependent manner without respiratory depression.^7^

Introduced primarily as an alternative to propofol or benzodiazepines, dexmedetomidine has also shown promising potentials in preventing postoperative delirium^8^ presumably because of its γ-aminobutyric acid receptor-sparing activity.^9^ However, a meta-analysis could not find significant effect in delirium risk reduction with dexmedetomidine.^10^ On the other hand, a recent systematic review found promising potentials of dexmedetomidine in this regard.^11^ In order to further refine the present day evidence, this study systematically reviewed and meta-analyzed the randomized clinical trials (RCTs) that utilized dexmedetomidine with general anesthesia perioperatively or as ICU sedation and assessed postoperative/postinfusion neurocognitive function by using a reliable cognitive assessment test.

## METHODS

### Ethical Statement

All analyses were based on previous published studies, thus no ethical approval and patient consent are required.

### Literature Search

The literature search was undertaken across several electronic databases including EBSCO, Embase, Google Scholar, Ovid SP, PubMed, Scopus, and Web of Science. The major MeSH and other keywords—dexmedetomidine, analgesia, anesthesia, surgery, perioperative, postoperative, intraoperative, premedication, cognitive dysfunction, cognition, neurocognitive, brain function, delirium, emergence agitation, minimental state examination (MMSE), digital symbol substitution test (DSST), adapted cognitive examination, confusion assessment method for ICU (CAM-ICU), randomized trial, clinical trial, etc—were used in different logical combinations and phrases. The search encompassed original research articles published between 1985 and 2014.

### Inclusion and Exclusion Criteria

The inclusion criteria were as follows: studies with medical/surgical/ICU patients or that used dexmedetomidine experimentally to healthy individuals in order to examine the effects of dexmedetomidine on the incidence of neurocognitive dysfunction in the postanesthesia period; used suitable controls/comparators; and utilized a valid neurocognitive assessment tool to diagnose and measure the neurocognitive function and provided the incidence of the neurocognitive dysfunction as number of events. Exclusion criteria were as follows: studies examining mental state by means other than the use of a neurocognitive assessment tool; studies examining the effects of dexmedetomidine on memory; case reports or case series; and studies with relevant but inadequate information for the meta-analysis of risk differences (RDs).

### Quality Assessment of Trials

The Cochrane Collaboration Risk of Bias Assessment Tool for the assessment of RCTs^12^ was used for the quality assessment of the randomized controlled trials included in this meta-analysis. This tool examines internal validity of the trial, and risk of bias in various phases of trial conduct and outcome analyses. Each of the individual studies was also thoroughly evaluated with respect to study design, methodology, outcome dissemination and interpretation, and strengths and limitations.

### Data Extraction, Synthesis, and Statistical Analysis

Important information including outcome measures, anesthetic dosage and usage, surgery type and duration, and participants’ demographic characteristics were obtained from identified articles and synthesized on datasheets for use in the meta-analyses by 2 researchers independently. Interrater reliability was good (Cohen κ = 0.95).

Meta-analyses were carried out with the RevMan software (Version 5.2; The Cochrane Collaboration, 2008) under fixed effects model as well as random effects model (REM). Neurocognitive dysfunction events presented in the individual studies were used to calculate the RDs between dexmedetomidine-treated and control or comparator-treated patients and then an overall effect was generated which was a weighted average of the inverse variance adjusted effect sizes of individual studies (RD, along with 95% confidence interval).

Statistical heterogeneity between studies was tested by *I*^2^ index. Sensitivity analyses were performed, wherever necessary. Visual examinations of the asymmetry of the funnel plots were performed as a proxy measure of the rough assessment of selection biases, including publication bias.

Subgroup analyses were carried out in order to evaluate the impact of dose concentration, mode of dexmedetomidine administration, type of neurocognitive assessment test, and comparator type on the overall results. For each variable, subgroup pair was first defined and then meta-analyzed. The effect sizes of each member of a subgroup pair were subjected to χ^2^ test for examining the significance of difference.

## RESULTS

**FIGURE 1:**
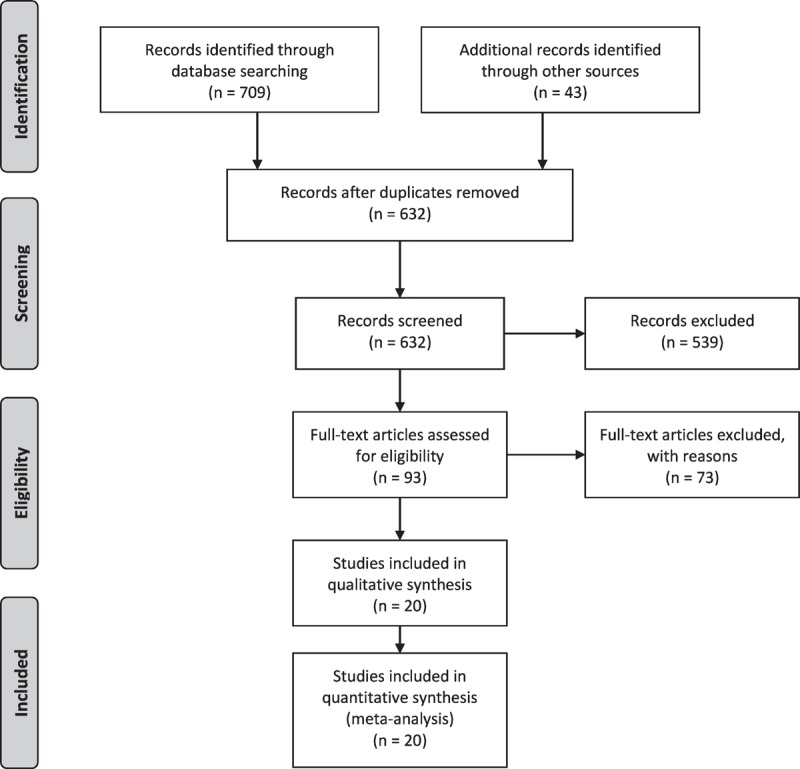
Flowchart of study screening and selection process.

Twenty studies^13–32^ were selected for the meta-analyses. A flowchart of the study screening and selection process is given as Figure 1. Data of 2612 patients and healthy individuals from the included studies are used for this meta-analysis. Demographic characteristics as mean ± standard deviation (range) of these individuals were age, 53 ± 9 (14–75) years; weight, 63 ± 7.5 (58–78) kg; and height, 165 ± 6.6 (155–177) cm. There were no significant differences between the comparative groups in the included studies with respect to age, weight, and height.

Major characteristics of the included studies relevant to the present study are presented in Table 1 and the quality assessment of the included studies is presented in Table 2. Quality of the included studies was moderate to good, in general. Selections biases including publication bias were also minimal as depicted by the funnel plot (Figure S1, http://links.lww.com/MD/A221).

**TABLE 1:**
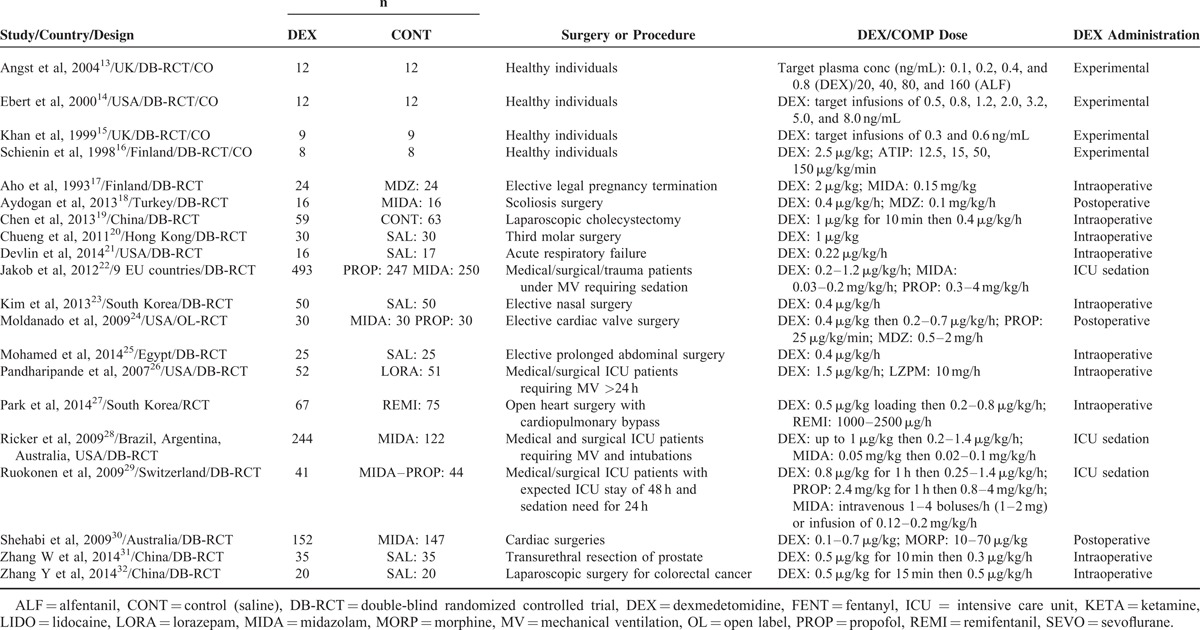
Characteristics of the Individual Studies

**TABLE 2:**
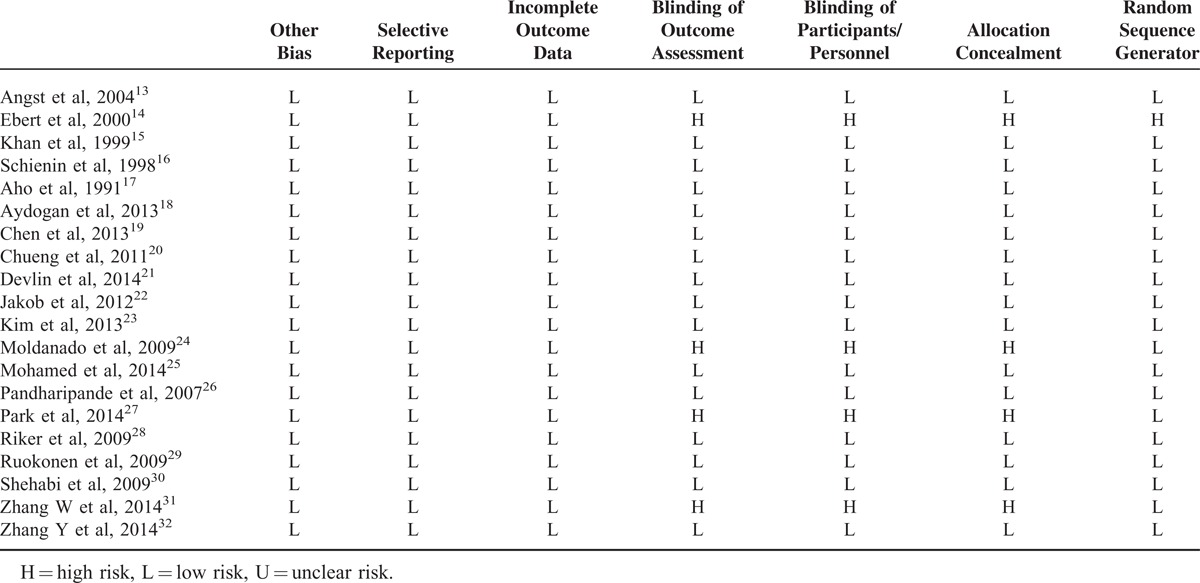
Risk of Bias Assessment in the Included Studies

Of the included studies, 4^13–16^ recruited healthy individuals in crossover designs, and 16^17–32^ recruited ICU medical/surgical patients. Mode of dexmedetomidine administration in the studies that recruited patients was intraoperative in 10, postoperative in 3, and ICU sedation in 3. In these studies, initial dose of dexmedetomidine (mean ± standard deviation) was 0.68 ± 0.27 (initial) and maintenance dose was 0.54 ± 0.32.

Neurocognitive assessment was carried out with CAM-ICU in 7, DSST in 4, and MMSE in 4 studies, and 1 study each utilized intensive care delirium screening checklist, trail making test, montreal cognitive assessment test, Stroop color word interference test, and sedation–agitation scores.

**FIGURE 2:**
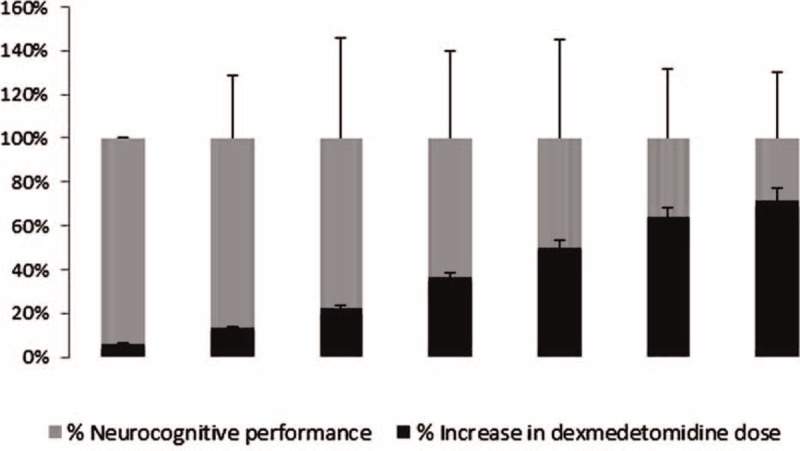
Synthesis of 3 studies^13,14,16^ that evaluated the dose–response relationship of dexmedetomidine. From left to right, percent increase in the dose parallels percent change from baseline in the performance of a neurocognitive test of the healthy volunteers.

Main findings of the meta-analysis are summarized in Table 1. Pooling of data from 3 studies with healthy individuals^13–15^ showed that dexmedetomidine treatment decline neurocognitive function in a dose-dependent manner (Figure 2) and 2 studies^16,17^ also demonstrated that dexmedetomidine antagonization with atipamezole can reverse neurocognitive decline in healthy individuals.

**FIGURE 3:**
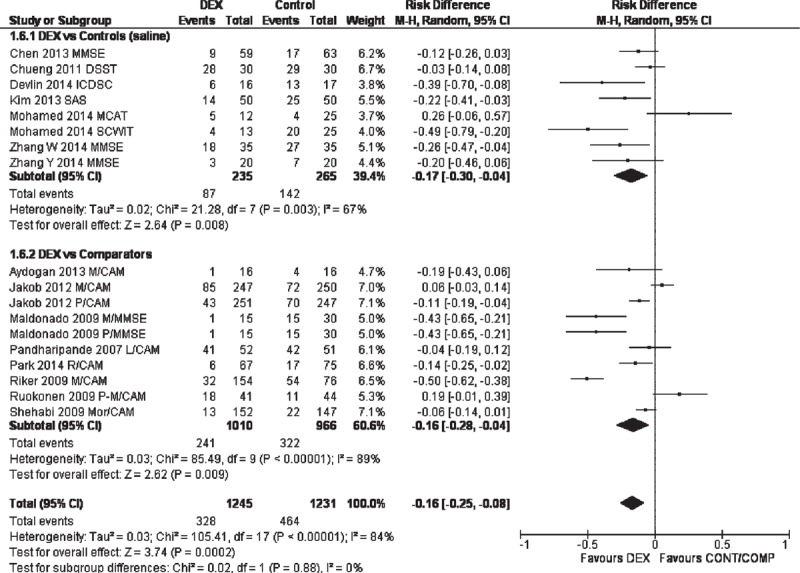
Forest graph showing the results of the meta-analyses of risk differences between dexmedetomidine and controls/comparators in the performance of a neurocognitive assessment test. Study identities follow comparator (L, lorazepam; M, midazolam; Mor, morphine; P, propofol; R, remifentanil) and neurocognitive assessment test name. CAM-ICU = cognitive assessment method for intensive care unit, DSST = digital symbol substitution test, ICDSC = intensive care delirium screening checklist, MCAT = Montreal cognitive assessment test, MMSE = minimental state examination, SAS = sedation–agitation score, SCWIT = Stroop color word interference test.

Dexmedetomidine treatment was associated with significantly lower risk of neurocognitive dysfunction in the postoperative/postanesthesia period. In the overall meta-analysis, RD (95%) was −0.16 (−0.25, −0.08); *P* = 0.0002; REM (Figure 3), whereas, it was −0.17 (−0.30, −0.04); *P* = 0.008; REM between dexmedetomidine and saline-treated patients and −0.16 (−0.28, −0.04); *P* = 0.009; REM between dexmedetomidine and comparator-treated patients.

**FIGURE 4:**
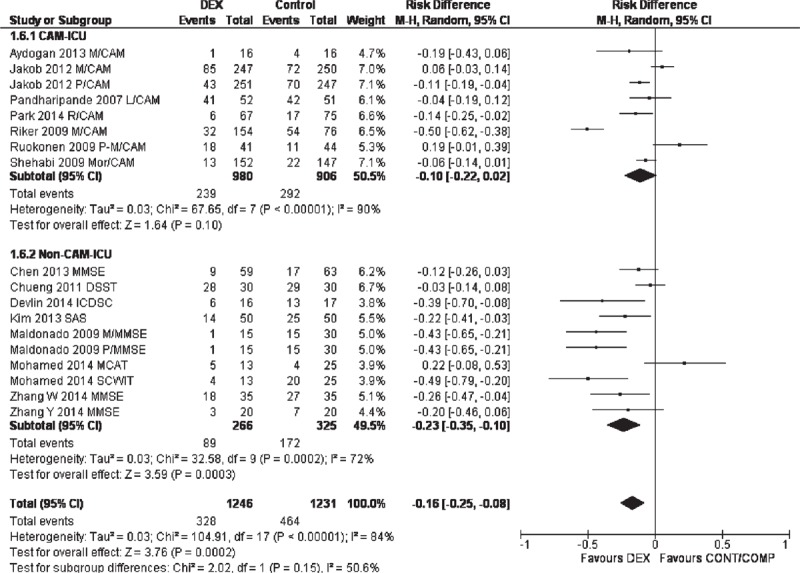
Forest graph showing the results of a subgroup meta-analysis of the studies that utilized CAM-ICU versus all other neurocognitive assessment tools. CAM-ICU = cognitive assessment method for intensive care unit, DSST = digital symbol substitution test, ICDSC = intensive care delirium screening checklist, MCAT = Montreal cognitive assessment test, MMSE = minimental state examination, SAS = sedation–agitation score, SCWIT = Stroop color word interference test.

**FIGURE 5:**
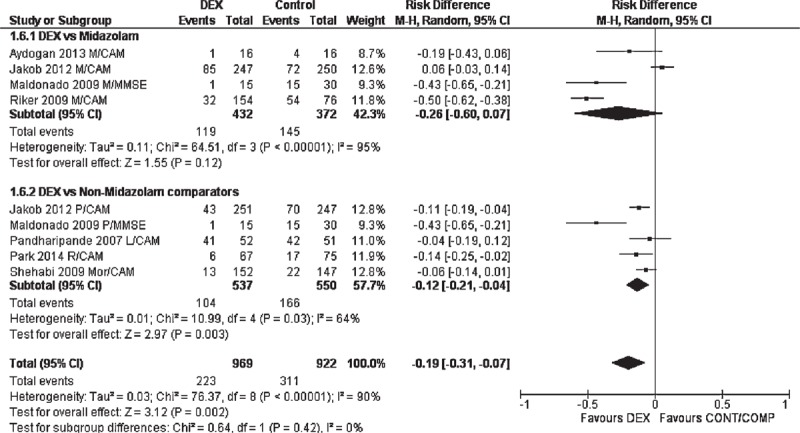
Forest graph showing the results of a subgroup meta-analysis of the studies that utilized midazolam versus all other comparators. CAM-ICU = cognitive assessment method for intensive care unit, DSST = digital symbol substitution test, ICDSC = intensive care delirium screening checklist, MCAT = Montreal cognitive assessment test, MMSE = minimental state examination, SAS = sedation–agitation score, SCWIT = Stroop color word interference test.

In the subgroup analyses, however, there was no significant difference between dexmedetomidine and control/comparators when studies with CAM-ICU only (RD: −0.10 (−0.22, 0.02); *P* = 0.1; REM; Figure 4) or midazolam as comparator only (RD: −0.26 (−0.60, 0.07); *P* = 0.12; REM; Figure 5) were meta-analyzed. Outcomes of other subgroup analyses are presented in Table 3.

**TABLE 3:**
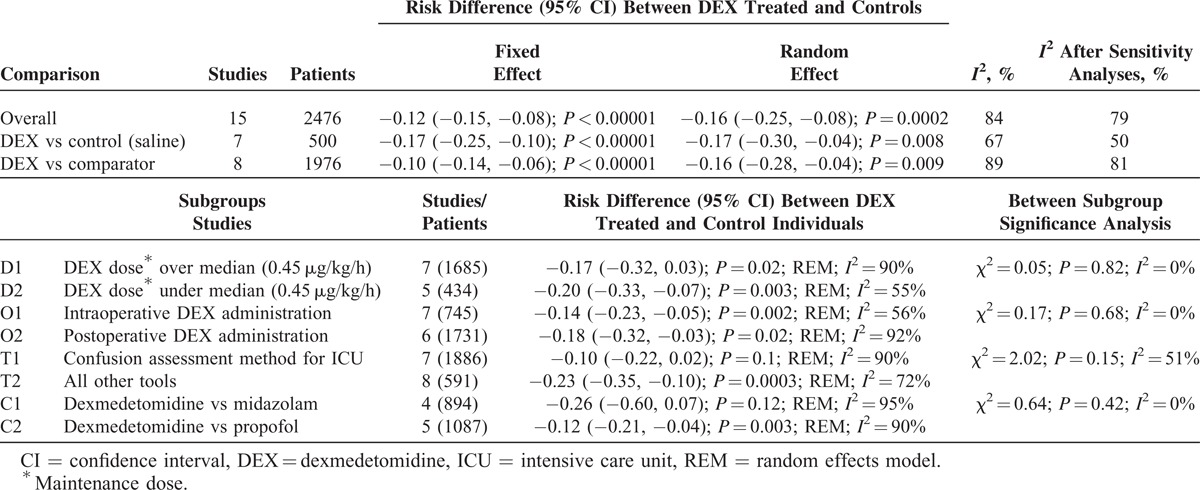
Outcomes of the Meta-Analyses and Subgroup Analyses

When the effect sizes of submeta-analyses were subjected to a χ^2^ test in order to test the between subgroup differences, there were no significant differences in the outcomes between the subgroup pairs—CAM-ICU versus MMSE neurocognitive assessment; midazolam versus propofol as comparators (Table 1; Figures 4 and 5); dexmedetomidine maintenance dose of equal to or above versus under median (0.425 μg/kg/h); and intraoperative versus postoperative/ICU-sedation dexmedetomidine administration (Table 1; Figures S2 and S3, http://links.lww.com/MD/A221 in supplementary material).

## DISCUSSION

This meta-analysis has revealed that dexmedetomidine use significantly reduces the risk of neurocognitive dysfunction in the postinfusion period in comparison with saline as well as with comparator anesthetics. However, in the subgroup analyses, a meta-analysis of 7 studies that utilized CAM-ICU for neurocognitive assessment, no significant difference between dexmedetomidine and comparators/controls-treated patients was found. Moreover, meta-analysis of 4 studies that used midazolam as comparator anesthetic also could not meet with any significance difference. These findings indicate that there can be some impact of the neurocognitive assessment method, dexmedetomidine dosage, and clinical heterogeneity on the overall outcomes of postoperative/postinfusion neurocognitive function as well as its assessment.

Some studies with relevant information could not be included in the present meta-analysis because of the eligibility criteria of the present study. Among these, Ji et al,^33^ who retrospectively analyzed the outcomes of over 1000 patients who underwent coronary artery bypass surgeries, could not find any significant difference in the incidence of neurocognitive events between dexmedetomidine-treated and control patients. These authors defined delirium as “illusions, confusion, and cerebral excitement in the postoperative period and having a comparatively short course.” In a similar retrospective analysis, Dasta et al^34^ also could not find any significant difference in the incidence of delirium between dexmedetomidine–propofol–midazolam-treated and only propofol–midazolam-treated patients where the diagnosis guidance was based on ICD-9-CM (International Classification of Diseases, 9th Revison, Clinical Modification, codes 292.81, 293.1). Martin et al^35^ against control, and Herr et al^36^ and Terao et al^37^ against propofol found no significantly different effects of dexmedetomidine in the incidence of confusion and agitation as adverse events.

Among others, Bekker et al^38^ in a RCT compared dexmedetomidine treatment with saline (both with propofol and fentanyl) in patients with major spinal surgery and found that MMSE scores dropped significantly from baseline on postoperative day 1 and a significant difference persisted between dexmedetomidine and saline-treated groups 3 days after surgery. Ohtsuka^39^ found that postoperative dexmedetomidine administration to elderly patients with cognitive impairment manifested beneficial effects in preventing neurocognitive dysfunction-related effects. Bustillo et al^40^ have reported that neurocognitive testing was not possible even at 1 hour after the cessation of infusion when they administered dexmedetomidine to individuals requiring interventional neuroradiologic procedures.

It has been opined that one possible mechanism of dexmedetomidine action can be its dose-sparing effects for other anesthetics such as lorazepam.^26^ It is well recognized that α-2 agonists especially dexmedetomidine possess anesthetic and analgesic-sparing effects.^41,42^ Moreover, synergistic effects of dexmedetomidine with benzodiazepines are also reported.^43^ In the trial of Jakob et al,^22^ although there was no significant difference in the incidence of neurocognitive dysfunction events in comparison with midazolam, dexmedetomidine administration led to significantly lower incidence of neurocognitive dysfunction when compared with propofol. On the other hand, dexmedetomidine has also been found to prevent sevoflurane-induced emergence agitation in children when administered 5 minutes before the end of surgery^44^ that shows that interactions with other drugs also play a role in manifesting effects of dexmedetomidine.

Several factors are needed to be taking into account while interpreting the results of trials examining efficacy of dexmedetomidine in postoperative neurocognitive function. Among these, the equivalence of dosing while using a comparator anesthetic^45,46^ and the outcome measure reliability^47^ are more important. In the present study, these factors might have also played role in determining the overall effect size as the statistical heterogeneity was higher. Moreover, there was disagreement in the results with different neurocognitive assessment tools. Studies have also shown that hypoactive delirium is more common than agitational delirium (61% vs 8%), but the identification of hypoactive delirium is difficult under normal neurocognitive tests.^48^

For this meta-analysis, neurocognitive dysfunction events on the first postoperative/postinfusion day were taken into account because of the less availability of data for later days. This is an important limitation. Clinical and methodological heterogeneity between the included studies may also have impact on the overall results that is also evident from statistical heterogeneity that was higher in the overall meta-analysis and some submeta-analyses.

## CONCLUSION

Dexmedetomidine treatment during perioperative conditions or as ICU sedation has been found to be associated with significantly better neurocognitive function of the patients, but factors such as neurocognitive assessment method, drug interactions, and clinical heterogeneity may have impacts on these results. Further studies are required to refine the evidence achieved herein.
